# Supplementary material for: Comprehensive Annotation and Functional Exploration of MicroRNAs in Lettuce
Source: Front Plant Sci. 2021 Dec 24;12:781836. doi: 10.3389/fpls.2021.781836 (PMC8739914; doi:10.3389/fpls.2021.781836)
Supplement: Supplementary Data 1 — Results of categories 0–2 from PARE-Seq analysis (including three subfiles:1_1, 1_2, 1_3). [file Data_Sheet_10.ZIP › GSM2230756.plot/Lsa-miR396a_Lsat_1_v5_gn_4_162180.1_516_TPlot.pdf]

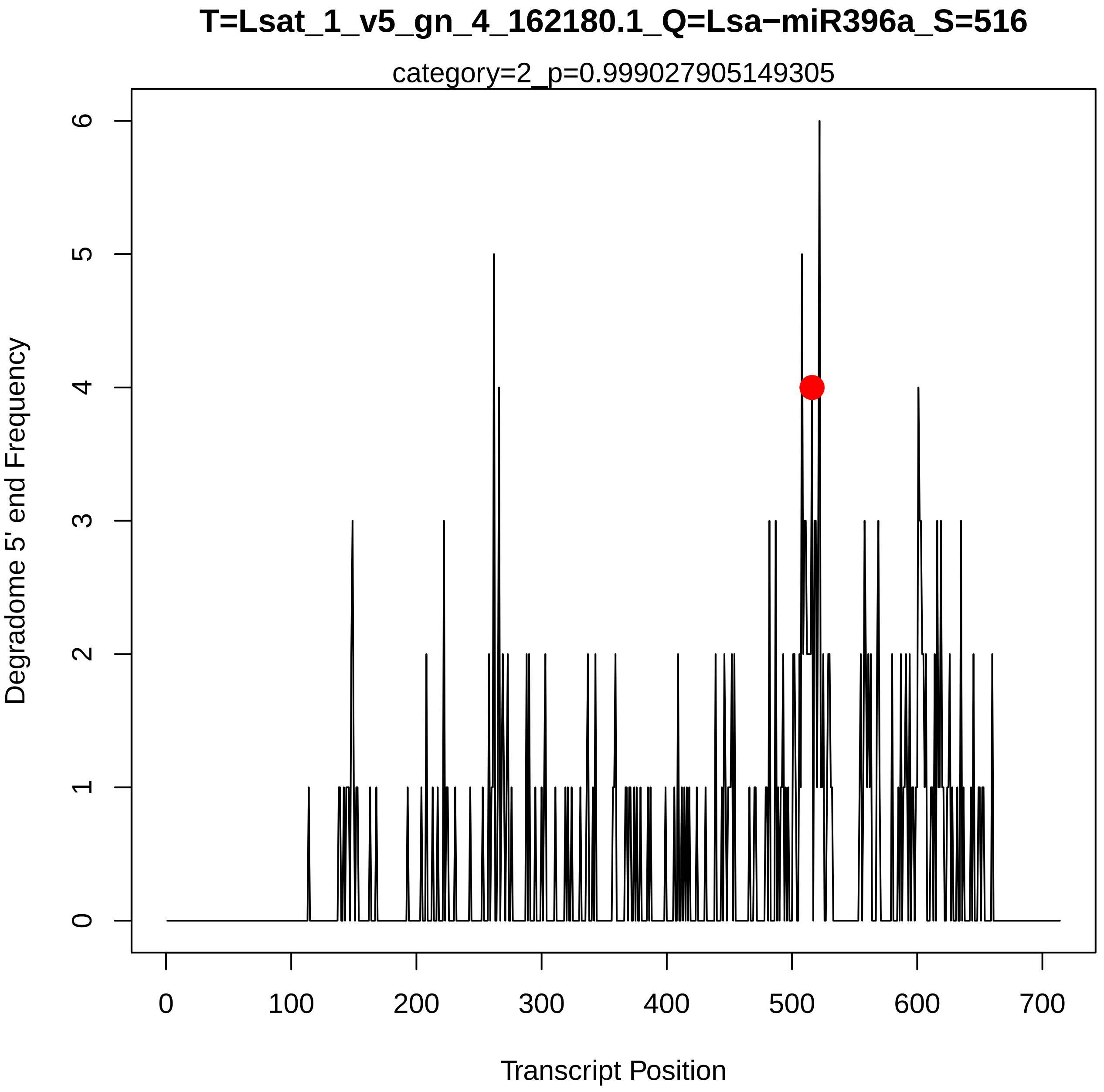
T=Lsat_1_v5_gn_4_162180.1_Q=Lsa−miR396a_S=516
category=2_p=0.999027905149305
Degradome 5' end Frequency
Transcript Position
0
1
2
3
4
5
6
0
100
200
300
400
500
600
700
